# HIV-1 virologic failure in the RESINA cohort: lessons from two decades of real-world data

**DOI:** 10.1007/s15010-025-02713-7

**Published:** 2025-12-17

**Authors:** Smaranda Gliga, Micha Böhm, Nadine Lübke, Alexander Killer, Falk Hüttig, Lila Haberl, Jörg Timm, Claudia Müller, Eva Heger, Joachim Büch, Gerd Fätkenheuer, Clara Lehmann, Mark Oette, Martin Hower, Heribert Knechten, Niels Schübel, Stefan Esser, Stephan Schneeweiß, Nazifa Qurishi, Katja Römer, Jürgen K. Rockstroh, Rolf Kaiser, Tom Luedde, Björn-Erik Ole Jensen

**Affiliations:** 1 Department of Gastroenterology, Hepatology and Infectious Diseases, Medical Faculty and University Hospital Düsseldorf, Heinrich Heine University Düsseldorf, Düsseldorf, Germany; 2 Institute of Virology, Faculty of Medicine and University Hospital of Cologne, University of Cologne, Cologne, Germany; 3 German Center for Infection Research (DZIF), Partner Site Bonn- Cologne, Cologne, Germany; 4 Institute of Virology, Medical Faculty and University Hospital Düsseldorf, Heinrich Heine University Düsseldorf, Düsseldorf, Germany; 5 Division of Infectious Diseases, Department I of Internal Medicine, Medical Faculty and University Hospital Cologne, University of Cologne, Cologne, Germany; 6 Krankenhaus Der Augustinerinnen, Cologne, Germany; 7 Klinikum Dortmund gGmbH, Hospital of University Witten/Herdecke, Dortmund, Germany; 8 Private Practice, Aachen, Germany; 9 Klinikum Osnabrück, Osnabrück, Germany; 10 Department of Dermatology and Venerology, Faculty of Medicine and University Hospital of Essen, University of Duisburg-Essen, Essen, Germany; 11 Private Practice Hohenstaufenring, Cologne, Germany; 12 Private Practice Gotenring, Cologne, Germany; 13 Department of Medicine I, University Hospital Bonn, Bonn, Germany

**Keywords:** HIV, Virologic failure, Drug resistance, Resuppression, COX regression, RESINA

## Abstract

**Purpose:**

To quantify virologic failure (VF), identify predictors, characterize resistance patterns at failure, and evaluate time to resuppression in the RESINA cohort.

**Methods:**

ART-naïve adults initiating ART in 2001–2024 were followed. VF was defined as at least one HIV-1 RNA > 200 copies/mL after suppression or ≥ 0.5-log₁₀ rebound. Participants were grouped by treatment era (2001–2007, 2008–2013, ≥ 2014), reflecting availability of drug classes. Genotypes at baseline and VF were interpreted using the HIV-GRADE algorithm. Predictors of VF were assessed with logistic regression; time to resuppression (< 50 copies/mL) after first VF with Cox models and Kaplan–Meier plots.

**Results:**

Among 5136 participants, 139 (2.7%) had VF; rates declined across eras (4.7%, 2.6%, 1.7%). Independent predictors were injection-drug use (OR 1.74), CD4 < 200/µL (OR 2.32), and ART start in 2001–2007 (OR 1.95); MSM acquisition was protective (OR 0.32). At failure, 36 patients showed resistance, often multiclass (61%); INSTI resistance was rare (n = 5). After first VF, 122/139 cases resuppressed (median 147 days). Male sex predicted faster resuppression (HR 1.81); higher failure VL trended to slower resuppression (HR 0.84 per log₁₀). INSTI-based switches consistently achieved resuppression in descriptive analyses and were not associated with multiclass resistance.

**Conclusion:**

VF was uncommon and declined over time, reflecting improved regimen potency and tolerability. Failures were associated with late presentation and IDU, consistent with adherence barriers. Resistance often involved multiple classes, while INSTI resistance remained infrequent. Early, genotype-guided optimization, preferably to INSTI-based therapy, combined with targeted adherence support may improve outcomes.

**Supplementary Information:**

The online version contains supplementary material available at 10.1007/s15010-025-02713-7.

## Introduction

The widespread introduction of combination antiretroviral therapy (cART) has transformed HIV from a fatal illness to a manageable chronic disease, dramatically improving both survival and clinical outcomes for affected individuals [1, 2]. In recent years, newer regimens featuring enhanced safety profiles, once-daily single-tablet formulations, and the approval of long-acting injectable agents have further simplified treatment and strengthened adherence [3–5]. Sustained adherence not only ensures durable viral suppression but is also associated with improved health-related quality of life (HR-QoL) [1].

Despite substantial therapeutic advances, virologic failure (VF) remains a significant challenge in HIV management. The principal drivers of VF include suboptimal adherence, preexisting resistance mutations, and viral subtype–specific characteristics that may accelerate rebound or increase the likelihood of resistance development [6–8]. Sociodemographic factors such as younger age, male sex, nonwhite ethnicity, migration from high-prevalence regions, injection drug use (IDU), and lower socioeconomic status have also been linked to VF, primarily through their impact on adherence, continuity of care, and access to treatment [9–13]. Clinical determinants including low baseline CD4 T-cell count, elevated HIV-1 RNA levels at initiation, and delayed diagnosis or late presentation consistently predict poorer outcomes [5, 11, 14, 15]. In addition, comorbid conditions, such as obesity, which is particularly relevant among individuals receiving long-acting injectable regimens, and structural barriers, including limited healthcare access in rural settings, may further increase the risk of VF [16–18].

Given these diverse contributors, understanding how resistance mutations evolve and persist is vital for informing treatment decisions, optimizing regimen selection and preventing ongoing viral replication. Previous studies have catalogued the range of resistance mutations that compromise cART efficacy and necessitate regimen switches [8, 19–21]. However, the extent to which specific resistance mutation patterns influence VF outcomes in real-world cohorts with limited prior treatment exposure remains unclear. The RESINA cohort provides a unique opportunity to address these gaps, as it systematically collects longitudinal virologic and clinical data on previously untreated individuals. This enables a detailed analysis of both the frequency and the clinical consequences of resistance-associated mutations.

In this analysis we aimed to quantify the frequency and determinants of VF, and to characterize the associated resistance mutations in a cohort of previously untreated participants with HIV from the RESINA cohort. As a secondary objective, we aimed to determine the proportion of participants who regained virologic resuppression after VF, and to identify the clinical and virologic factors associated with subsequent resuppression. Such insights are essential for optimizing first-line regimens, guiding timely regimen switches, and improving long-term HIV outcomes.

## Materials and methods

This analysis used data from the RESINA cohort, a multicenter observational study investigating transmitted HIV drug resistance in ART-naive individuals from North Rhine-Westphalia, Germany. We included all participants diagnosed with HIV and initiating ART between 2001 and 2024. Study entry was defined as the date of written informed consent and collection of the first study sample before ART, whose initiation varied according to treatment guidelines across the observation period. Late presentation/diagnosis of HIV was defined in this study as a CD4 cell count below 200 cells/µL at the time of HIV diagnosis. This differs from the European Centre for Disease Prevention and Control (ECDC) definition, which classifies late presentation as a CD4 count < 350 cells/µL or an AIDS-defining event within three months of diagnosis. [22]. We applied the stricter threshold of < 200 cells/µL to ensure a uniform classification across the cohort.

### Outcomes

The primary binary outcome was virologic failure (VF; ≥ 1 event vs none). VF was defined as at least one HIV-1 RNA measurement > 200 copies/mL occurring after prior viral suppression (< 50 copies/mL). In patients who did not achieve full suppression, VF was defined as at least one HIV-1 RNA measurement showing a ≥ 0.5-log₁₀ increase from the post-ART nadir following an initial decline, even if the nadir remained above the assay’s lower limit of quantification (LLQ).

Subsequent VF episodes were defined using the same virologic criteria (HIV-1 RNA > 200 copies/mL), provided they occurred after a documented resuppression (< 50 copies/mL).

Participants who never achieved viral suppression after ART initiation were defined as those who never had an HIV-1 RNA viral load < 50 copies/mL.

### Data collection

Clinical data included demographics, regimen, adherence (documented treatment interruption; missed ART-related appointments; descriptive only)**,** and HBV/HCV/HDV coinfections. Virologic data comprised plasma HIV-1 RNA (viral load (VL)) and the timing of genotypic resistance testing. Participants were stratified into three ART-initiation eras reflecting major guideline-driven shifts in first-line therapy and the availability of agents with higher genetic barriers to resistance in Germany: Group 1 (2001–2007; pre-darunavir/raltegravir), Group 2 (2008–2013; post-darunavir/raltegravir), and Group 3 (2014 onwards; widespread use of second-generation integrase inhibitors such as dolutegravir (DTG)), in line with evolving European and international HIV treatment guidelines [5, 23].

### Resistance testing

Genotypic resistance was assessed at study entry and again at the time of virologic failure (VF). Plasma HIV-1 RNA was amplified and sequenced using conventional Sanger assays from the start of the study and Illumina next-generation sequencing (NGS) from 2015 onward. Ten percent consensus FASTA files were generated for NGS data to ensure optimal comparability with earlier population-based Sanger sequences, based on internal validation tests; when RNA amplification was unsuccessful, paired proviral DNA from peripheral blood mononuclear cells was analyzed to avoid loss of resistance information [24]. Composite nucleotide sequences spanning the protease (PR), reverse transcriptase (RT), and integrase (IN) regions were interpreted with the HIV-GRADE algorithm [25], an independent rules-based resistance-interpretation system whose initial development was based on the 2003 Stanford HIVdb source code and later expanded using clinical datasets, published correlations, and geno2pheno bioinformatic models. Transmitted drug resistance (TDR) was assessed from baseline sequences using the HIV-GRADE mutation list, available within the HIV-GRADE HIV-1 tool [26].

To detect the subtype, we analyzed all sequences spanning the protease (PR) and reverse transcriptase (RT) regions available for that patient using the HIV-GRADE interpretation system and the geno2pheno-subtype tool for the newer sequences.

All major and accessory mutations were cataloged across nucleos(t)ide and non-nucleoside RT inhibitors, protease inhibitors and integrase-strand-transfer inhibitors (INSTI), with particular attention to the canonical mutation pathways that underlie the stepwise evolution of raltegravir resistance described by Sichtig et al [19].

### Statistical analysis

All analyses were conducted in SPSS v27 (IBM Corp., Armonk, NY). Baseline was defined as study entry. Descriptive statistics (frequencies/percentages; median/range) summarized characteristics and mutation distributions; group comparisons used t-tests/ANOVA or Mann–Whitney/Kruskal–Wallis as appropriate.

To identify predictors of VF, we fit a cohort-wide binary logistic regression model including all 5,136 participants, restricted to one record per individual (VF = 1 if ≥ 1 VF occurred during follow-up; VF = 0 otherwise). Covariates were prespecified based on prior evidence, biological plausibility, and data completeness. Age was modeled in 10-year increments. Sex was coded as male versus female/other. Route of acquisition was represented by two dummy variables for MSM and IDU (1 = yes, 0 = no), with heterosexual contact as the reference group. Baseline CD4 count was categorized as < 200 cells/µL, ≥ 200 cells/µL, or missing (reference), and baseline HIV-1 RNA as < 100,000 copies/mL, ≥ 100,000 copies/mL, or missing (reference). As a sensitivity analysis, an alternative CD4 threshold of 350 cells/µL, commonly used in European HIV surveillance reporting, was evaluated using an otherwise identical model specification, while retaining missing CD4 values as a separate category. In addition, a baseline HIV-1 RNA was examined using a ≥ 500,000 copies/mL cut-off. Year of ART initiation was grouped into 2001–2007, 2008–2013, and ≥ 2014 (reference). This specification yielded 10 predictors for 139 VF events, remaining within accepted limits to avoid model overfitting. To further assess the robustness of the model, we conducted additional multivariable logistic regression models extending the core analysis. Each supplementary model included one additional covariate of interest: HIV-1 subtype (B vs non-B), region of origin (Western Europe vs other), or TDR (any vs none). Because adherence ascertainment was inconsistent and frequently missing, this variable was used for descriptive summaries only and was not included in multivariable models. All models were restricted to the first VF episode per patient and adjusted for the same set of demographic, clinical, and treatment-era variables as in the core model.

For time to virologic resuppression after the first VF event, we used a Cox proportional hazards model including age, sex, presence of resistance mutations at time of VF, regimen switch at failure, log₁₀ HIV-1 RNA at failure, and HIV-1 subtype B. Patients lost to follow-up before resuppression were censored at the date of their last available HIV-1 RNA measurement; deaths prior to resuppression were censored at the date of death. We did not model death as a competing risk because the number of deaths was small and the primary endpoint was time to resuppression.

For logistic regression, we report odds ratios (ORs) with 95% confidence intervals (95% CI) and two-sided p-values (Wald tests). For Cox models, we report hazard ratios (HRs) with 95% CI and two-sided p-values (Wald). All tests were two-sided with α = 0.05. p-values are shown up to three decimals; values < 0.001 are reported as p < 0.001.

Kaplan–Meier curves stratified by sex were used to visualize time to resuppression, with differences assessed by the log-rank test.

### Ethical considerations

The study protocol was reviewed and approved by the Ethics Committee of the Heinrich Heine University (Study No. 4862), and all participants provided written informed consent according to the Declaration of Helsinki.

## Results

### Baseline characteristics and rates of virologic failure

**Fig. 1:**
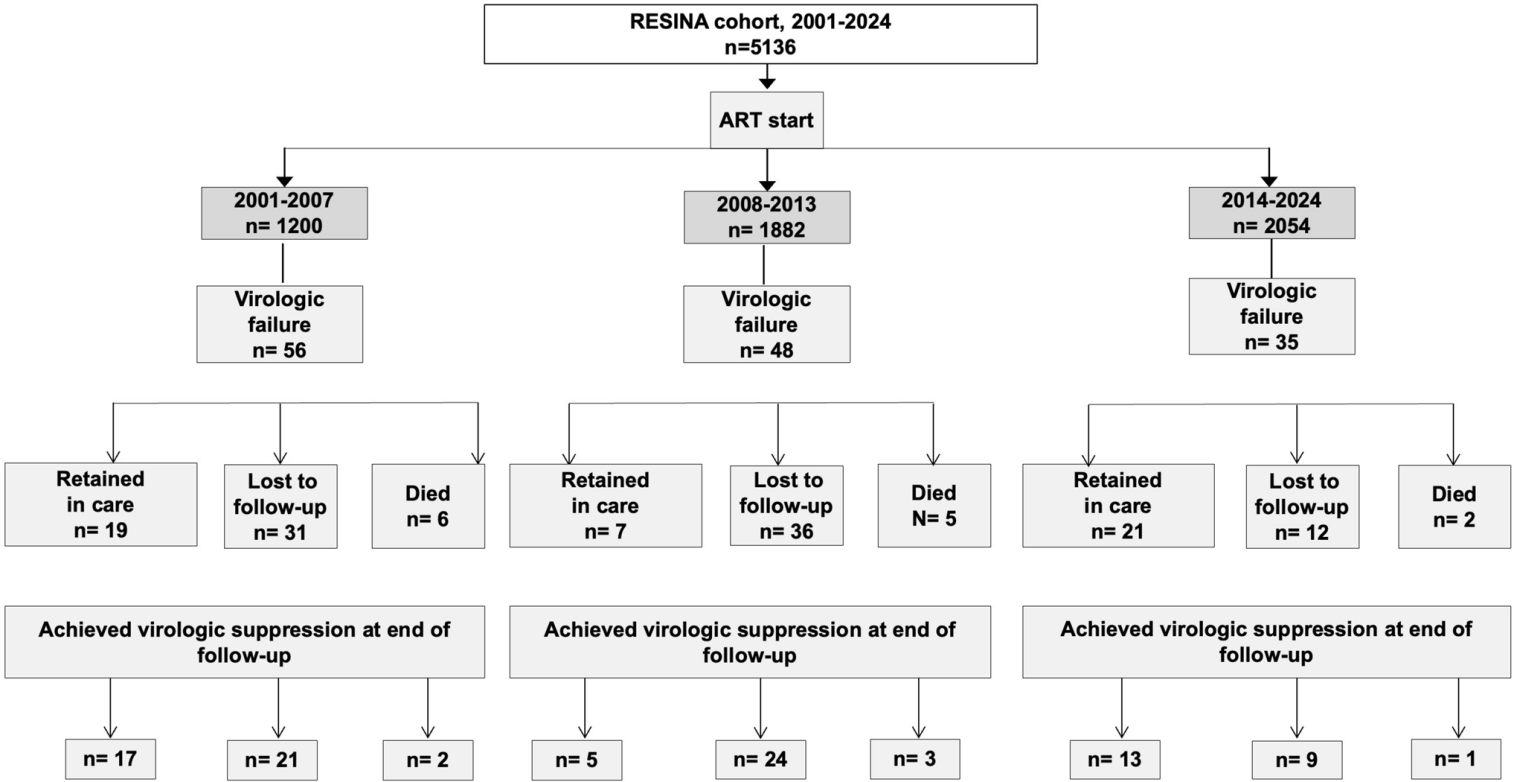
Patient flow through the RESINA cohort (2001–2024), from ART initiation to first virologic failure and final suppression status.Of 5136 ART‐treated participants, those with first virologic failure are shown by calendar period (2001–2007, 2008–2013, 2014–2024), then by disposition (retained in care, lost to follow-up, died) and by whether they achieved virologic suppression by end of follow-up. *n* number

Out of the 5136 patients included in the RESINA cohort, 139 experienced VF, corresponding to an overall VF rate of 2.7%. The patient flow diagram is presented in Fig. 1, and baseline characteristics by VF status are summarized in Table 1.

**Table 1:** Baseline characteristics of patients in the RESINA cohort according to virologic outcome (without vs. with virologic failure)

Group	No VF	VF	p-value
Number of patients (n)	4997	139	
Age (median, range)	39 (18–82)	37 (18–75)	
Age categories (n, %)			0.53
< 30 years	946 (18.9)	27 (19.4)	
30–39 years	1647 (33)	51 (36.7)	
40–49 years	1426 (28.5)	42 (30.2)	
50–59	724 (14.5)	12 (8.6)	
≥ 60 years	248 (5)	7 (5)	
Unspecified	6 (0.1)		
Gender (n, %)			
Male	4104 (82.1)	91 (65.5)	< 0.0001
Female at birth	869 (17.4)	48 (34.5)	
Other/unspecified	24 (0.5)		
Acquisition route (n, %)			
Heterosexual	976 (19.5)	42 (30.3)	< 0.0001
Heterosexual from high prevalence region	413 (8.3)	32 (23)	
MSM	2720 (54.4)	32 (23)	
IDU	270 (5.4)	18 (12.2)	
Other/unknown	618 (12.4)	15 (11.5)	
Region of origin (n, %)			
German	3276 (65.5)	79 (56.8)	< 0.0001
European (other)	118 (2.4)	9 (6.5)	
Sub Saharan Africa	258 (5.2)	24 (17.3)	
Asia	115 (2.3)	11 (7.9)	
Other/unknown	1230 (24.6)	16 (11.5)	
Median CD4 (range) at ART initiation cells/μL	273 (0–2006)	85 (1–484)	
< 200	945 (18.9)	60 (43.2)	< 0.0001
200–349	557 (11.1)	15 (10.8)	
≥ 350	963 (19.3)	6 (4.3)	
Missing	2532 (50.7)	58 (41.7)	
Median HIV-1 RNA (range) at ART initiation log_10_	4.6 (1.3–7)	4.8 (2.1- 6.3)	
< 100.000	2693 (53.9)	63 (45.3)	0.005
≥ 100.000	1326 (26.5)	54 (38.8)	
Missing	978 (19.6)	22 (15.9)	
HIV-1 Subtype (n, %)			
B	2927 (58.6)	76 (54.7)	< 0.0001
CRF02_AG	273 (5.4)	21 (15.1)	
C	129 (2.6)	8 (5.8)	
A1	194 (3.9)	7 (5)	
A6	130 (2.6)	4 (2.9)	
Other	1344 (26.9)	23 (16.5)	
Year of ART initiation			
2001–2007	1144 (22.9)	56 (40.3)	< 0.0001
2008–2013	1834 (36.7)	48 (34.5)	
≥ 2014	2019 (40.4)	35 (25.2)	
Transmitted drug mutations (TDR)			
Any mutation	357 (7.1)	8 (5.8)	0.62
None	4640 (92.9)	131 (94.2)	
Late diagnosis/late presenters (n, %)	945 (18.9)	60 (43.2)	< 0.0001
Deaths from any cause (n, %)	202 (4)	13 (9.4)	0.004

Values are given as number (%) unless otherwise stated. Age, CD4 and HIV-1 RNA are presented as median (range)

*VF* virologic failure, *MSM* men who have sex with men, *IDU* intravenous drug use, *TDR* transmitted drug resistance

Patients with VF were slightly younger (median age 37 years, range 18–75 vs. 39 years, range 18–82), and a lower proportion were male compared with patients without VF (65.5% vs. 82.1%, *p* < 0.001). Acquisition routes differed markedly: in the VF group, heterosexual transmission (including high-prevalence regions) and IDU were more common (30.3% vs. 19.5% and 12.2% vs. 5.4%, respectively), whereas men who have sex with men (MSM) transmission predominated among patients without VF (54.4% vs. 23%, *p* < 0.0001).

Regarding region of origin, 57% of VF patients originated from Germany, compared with 66% without VF. Sub-Saharan Africa was more frequently represented in the VF group (17.3% vs. 5.2%, *p* < 0.0001). Patients with VF initiated ART at lower CD4 cell counts (median 85/µL vs. 273/µL; < 200 cells/µL in 43.2% vs. 18.9%) and higher HIV-1 RNA levels (≥ 100,000 copies/mL in 38.8% vs. 26.5%, *p* = 0.005). Late presentation was significantly more frequent among VF patients (43.2% vs. 18.9%, *p* < 0.0001).

In a sensitivity analysis examining very high baseline HIV-1 RNA levels (≥ 500,000 copies/mL), 11 of 139 patients with VF (7.9%) and 513 of 4997 patients without VF (10.3%) had viral loads above this threshold (p = 0.45).

HIV-1 subtype B remained the most common subtype in both groups but was less predominant among VF patients (54.7% vs. 58.6%), with higher proportions of CRF02_AG (15.1% vs. 5.4%) and subtype C (5.8% vs. 2.6%, *p* < 0.0001). VF occurred more often in earlier treatment eras, with 40.3% of VF patients starting ART before 2007, compared to 22.9% in the no-VF group (*p* < 0.0001). TDR was uncommon overall and was observed in 7.1% (357/4997) of individuals without virologic failure compared with 5.8% (8/139) of those who experienced virologic failure (p = 0.62).

Overall mortality was higher in the VF group (9.4% vs. 4%, *p* = 0.004). Although more than half of VF patients (79/139) were lost to follow-up at some point, the median follow-up time was 9 years (range 1–24), and 85% (118/139) were followed for at least 5 years.

**Fig. 2:**
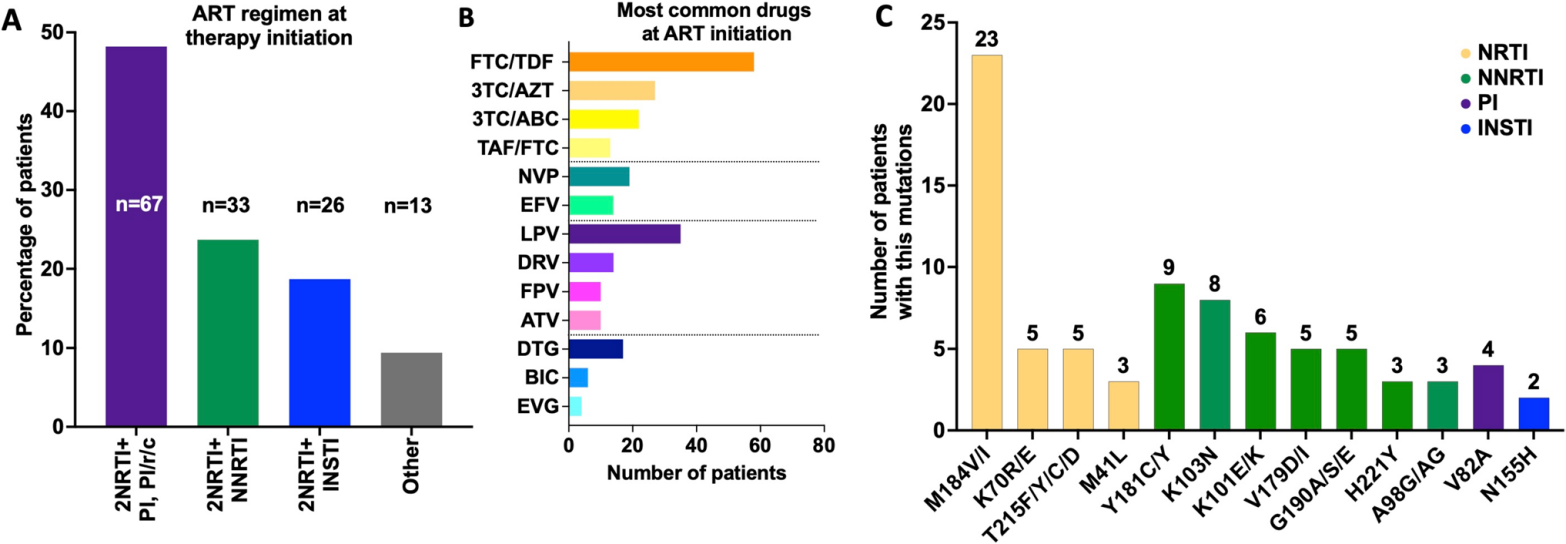
ART Regimens at treatment Initiation and resistance mutation frequencies in patients with VF. **A** Distribution of first-line ART regimen classes among individuals who later experienced VF. Bars show the percentage of patients in each regimen class, with absolute numbers (n) displayed on the bars. **B** Absolute number of patients initiating therapy with the most frequently used individual antiretroviral drugs. **C** Prevalence of major resistance-associated mutations detected at the time of VF, grouped by drug class (NRTI, NNRTI, PI, INSTI). Bars represent the number of patients harboring each mutation, and colors correspond to drug classes

At ART initiation, the most common regimens in the VF group combined two NRTIs, usually TDF/FTC, with either an NNRTI (most often nevirapine or efavirenz) or a boosted PI (most frequently lopinavir or darunavir) (Fig. 2). More than half of VF cases (77/139, 55.4%) occurred under the first ART regimen, and 34/139 (24.5%) occurred during the second. First VF/rebound occurred after a median of 608 days on ART (range 59–5186).

VF rates by treatment era were: group 1, 4.7% (56/1200); group 2, 2.6% (48/1882); and group 3, 1.7% (35/2054). Almost half (64/139) experienced a single VF event during follow-up, 21% (29/139) had two, and the remainder had three or more.

In a cohort-wide binary logistic regression model restricted to the first VF episode per patient, three covariates emerged as independent risk factors for VF: IDU (OR 1.74, 95% CI 1.00–3.00, *p* = 0.048), CD4 < 200 cells/µL at ART initiation (OR 2.32, 95% CI 1.56–3.45, *p* < 0.001), and ART initiation during 2001–2007 (OR 1.95, 95% CI 1.24–3.06, *p* = 0.004) (Table 2). This association between low CD4 count and VF remained consistent in sensitivity analyses using a higher CD4 threshold of 350 cells/µL (CD4 < 350: OR 1.88, 95% CI 1.29–2.72).

**Table 2:** Multivariable logistic regression of risk factors for virologic failure

Variable	OR	95% CI	p-value
Age (per 10 years)	0.88	0.75–1.04	0.14
Male sex (vs female/other)	0.80	0.53–1.20	0.29
MSM (vs heterosexual)	**0.32**	0.20–0.50	**< 0.001**
IDU (vs heterosexual)	**1.74**	1.00–3.00	**0.048**
CD4 < 200 cells/µL	**2.32**	1.56–3.45	**< 0.001**
CD4 ≥ 200 cells/µL	0.64	0.38–1.07	0.087
HIV-1 RNA < 100,000 copies/mL	0.98	0.58–1.68	0.95
HIV-1 RNA ≥ 100,000 copies/mL	1.38	0.81–2.36	0.24
ART start 2001–2007	**1.95**	1.24–3.06	**0.004**
ART start 2008–2013	1.20	0.75–1.92	0.44
ART start ≥ 2014	1.0 (ref)	–	–

Bold values indicate statistical significance (*p* < 0.05)

Reference categories: female/other sex, heterosexual acquisition, missing CD4, missing HIV RNA, ART start ≥2014

*OR* odds ratio, *CI* confidence interval, *MSM* men who have sex with men, *IDU* intravenous drug use. N=5130, VF events=139

In sensitivity analyses examining very high baseline HIV-1 RNA levels, very high baseline viremia (≥ 500,000 copies/mL) was not independently associated with VF (OR 0.64, 95% CI 0.27–1.54).

By contrast, acquisition through MSM contact was associated with a lower risk of VF (OR 0.32, 95% CI 0.20–0.50, *p* < 0.001). Other factors, including age, sex, baseline VL, and ART initiation during 2008–2013, were not significantly associated with VF. In supplementary models, neither HIV-1 subtype, TDR nor region of origin was independently associated with VF. Adjustment for subtype attenuated the effect of ART initiation during 2001–2007, indicating partial confounding by subtype distribution (Supplementary Tables 1a–c).

Adherence data were inconsistently recorded; among participants with VF, 83/139 (59.7%) had documented non-adherence (treatment pause and/or missed appointment). We did not evaluate adherence in adjusted models.

### HIV resistance mutations and cross-resistance patterns in the VF group

TDR mutations in HIV were identified in eight patients with VF, while acquired resistance mutations emerged in 28 during follow-up. Of those with acquired mutations, 11 had single-class resistance and 17 (61%) showed cross-resistance to two or more drug classes (Supplementary Table 2).

The most frequently observed NRTI resistance mutation was M184V/I (17 M184V and six M184I) detected in 23 patients (two transmitted, 21 acquired), followed by K70R/E in five patients (one transmitted, four acquired). Among NNRTI mutations, Y181C/Y occurred in nine patients (one transmitted, eight acquired), K103N in eight patients (two transmitted, six acquired), and K101E/K in six patients (all acquired). PI resistance was uncommon (n = 6), with V82A in four patients (one transmitted, three acquired). INSTI resistance (all acquired, no baseline INSTI TDR) was identified in five patients: N155H (n = 2), Y143C/R/S (n = 1), E92Q (n = 1), and R263K + G118R (n = 1). The latter pattern reflects dolutegravir resistance and has been described previously [27].

Supplementary Table 2 highlights extensive cross-resistance in several patients (e.g., M184V + T215Y + K103N + V82A conferring NRTI, NNRTI, and PI resistance).

Fourteen patients with transmitted or acquired mutations that do not impair cabotegravir (CAB) or rilpivirine (RPV) susceptibility would still be eligible to receive injectables (CAB/RPV LA), which might be an option to improve future adherence.

### Treatment outcomes post-failure in the VF group

Of 139 participants with any VF, 44 (31.7%) were unsuppressed at last follow-up (Fig. 1). After the first VF, 122 (87.8%) ever achieved resuppression, while 17 (12.2%) never resuppressed (HIV-1 RNA ≥ 50 copies/mL). Median time to resuppression was 147 days (range 13–2,015). The higher end-of-follow-up count reflects patients who later failed again or ended follow-up unsuppressed.

Of the total of 139 patients experiencing VF, 74 patients remained on the same drug class after VF (predominantly PI-based regimens, but also some NNRTI-, NRTI-only, and INSTI-based therapies), while 65 were switched to a different drug class. Common switches were PI → PI (n = 23), PI → INSTI (n = 14) and PI → other (n = 20); fewer moved NNRTI → PI (n = 6) or NNRTI → INSTI (n = 6), NRTI-only → PI (n = 5), and other → PI/INSTI (n = 6/2). Switches to INSTI-based regimens (PI → INSTI 3/3, NNRTI → INSTI 4/4, other → INSTI 7/7) and NRTI-only → PI (6/6) were the most consistently successful, whereas remaining on PI regimens (16/22, 73%) or switching NNRTI → PI (4/6, 67%) showed lower rates of re-suppression, and NRTI-only → NNRTI was least effective (1/2, 50%).

**Fig. 3:**
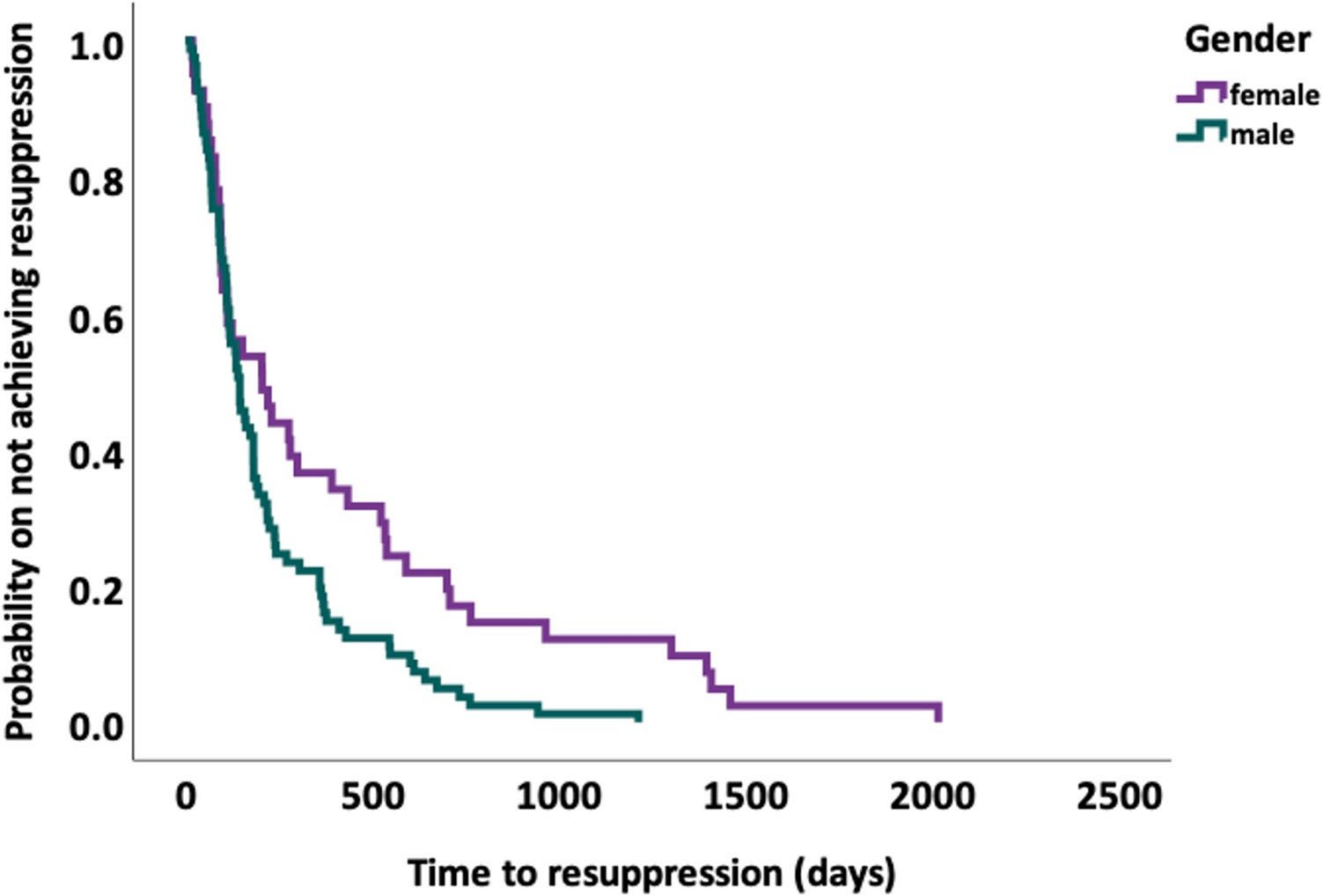
Kaplan–Meier estimates of time to virologic resuppression, stratified by gender. Kaplan–Meier survival curves showing the probability of remaining unsuppressed (HIV-1 RNA ≥ 50 copies/mL) over time after first virologic failure, stratified by sex. The green line represents male participants (n = 92), and the purple line represents female participants (n = 47). Censoring (patients lost to follow-up or without resuppression by study end) is indicated by tick marks. The log-rank test comparing the two curves yielded p = 0.009

Patients who failed to resuppress had significantly higher VL at failure compared with those who did resuppress (mean log₁₀ VL 4.7 vs. 3.8, *p* = 0.004). In multivariable analysis, male sex was independently associated with faster re-suppression (HR 1.81, 95% CI 1.15–2.86, *p* = 0.011), a finding also reflected in Kaplan–Meier curves (log-rank *p* = 0.009; Fig. 3). Regimen switch at failure showed a non-significant trend toward faster resuppression (HR 0.72, 95% CI 0.49–1.05, p = 0.087), while higher VL at failure trended toward slower resuppression (HR 0.84 per log₁₀ increase, 95% CI 0.71–1.00, *p* = 0.056). Age, resistance-mutation status at the time of VF, and subtype B were not significantly associated with resuppression. Overall, these results indicate that, once switch strategy is considered, male sex and lower VL at failure are the strongest predictors of faster resuppression, whereas age, resistance-mutation status, and subtype B appear to have little influence (Table 3).

**Table 3:** Cox proportional‑hazards regression of predictors for time to virologic resuppression after first virologic failure

Predictor	HR	95% CI	p-value
Age (per 10 years)	1.10	0.90–1.34	0.33
Sex (male vs. female)	1.81	1.15–2.86	**0.011**
Resistance mutations (yes/no)	0.89	0.59–1.37	0.62
Regimen switch (yes/no)	0.72	0.49–1.05	0.088
Log₁₀VL at failure	0.84	0.71–1.00	**0.056**
Subtype B vs non-B	0.93	0.61–1.41	0.72

Bold values indicate statistical significance (*p* < 0.05)

Cox proportional‑hazards model of time (days) from first virologic failure to confirmed re‑suppression, adjusted for age, sex, presence of resistance mutations, regimen switch at failure, VL at VF and HIV‑1 subtype B

*HR* hazard ratio, *CI* confidence interval

## Discussion

In this large, multicenter cohort of ART-naïve patients followed for more than two decades, VF was uncommon overall (2.7%) and declined markedly across calendar eras, from nearly 5% in the early 2000s to below 2% in the most recent decade. This decline reflects the impact of increasingly potent and tolerable regimens, particularly INSTI–based therapy, which has demonstrated superior effectiveness and durability compared with NNRTI- or PI-anchored regimens. These findings align with clinical trial and cohort data confirming faster suppression and improved durability under INSTI-based regimens [3, 4, 28].

Independent predictors of VF in our cohort, IDU and late presentation** (**CD4 < 200/µL), point to adherence and structural barriers rather than intrinsic regimen limitations. Evidence consistently links active substance use to suboptimal adherence and worse virologic outcomes, while opioid substitution therapy improves ART uptake, adherence, and suppression; this likely explains a sizable share of the excess VF we observed in people who inject drugs [29]. Similarly, late diagnosis is repeatedly associated with delayed virologic control and higher failure risk in European cohorts; our signal is aligned with those reports and with contemporary European cohort data [14, 15].

The higher VF in the earliest era also reflects historical context (less potent backbones, lower resistance barriers) and a different subtype mix; adjusting for subtype attenuated that effect in our sensitivity analyses, consistent with era- and subtype-related differences described elsewhere [15].

At failure, the pattern in our cohort, M184V and M184I among NRTIs and K103N/Y181C among NNRTIs, matches long-standing pathways seen with TDF/FTC + NNRTI backbones and explains much of the NNRTI cross-resistance we recorded [8].

Resistance to PIs and INSTIs was rare; we observed only isolated major INSTI mutations and a single case with DTG-associated changes, consistent with reviews showing that emergent DTG resistance remains uncommon but does occur under ongoing viremia [30]. As LA CAB/RPV use was not documented in the cohort at the time of data extraction, and treatment updates after loss to follow-up were incomplete, we could not assess VF outcomes related to injectable therapy in RESINA. However, real-world cohorts and Phase 3 trials consistently demonstrate high virologic success with CAB/RPV LA when administered on schedule, with failures largely associated with delayed dosing or baseline RPV resistance [31, 32].

Notably, TDR was not associated with higher VF risk here. This is biologically plausible: several common TDR mutations carry fitness costs and tend to revert in the absence of drug pressure, blunting clinical impact when potent modern regimens are used [33]. Many patients exhibited multiclass resistance (e.g., concurrent NRTI, NNRTI, and PI mutations), underscoring the need to favor INSTI-based regimens and, when necessary, newer antiretroviral classes to secure durable suppression in complex resistance profiles [20, 21, 34]. In addition, in our cohort, baseline resistance testing was systematically performed and considered when selecting initial therapy. This strategy likely prevented the use of compromised regimens, particularly among individuals with documented TDR, who were typically started on regimens with higher genetic barriers to resistance, and underscores the importance and effectiveness of baseline resistance screening in routine care.

Post-failure outcomes were generally favorable: 87.8% achieved at least one resuppression after the first VF (median 147 days). Two signals from our models mirror external data: higher VL at failure was linked with slower re-suppression, and switching therapy trended toward faster control, both consistent with reports that viral burden at switch predicts time-to-suppression and that timely regimen change improves outcomes. Current guidelines likewise recommend prompt reassessment (adherence + genotype) and early switch once VF is confirmed [5]. Prompt switch after confirmed VF is therefore prudent to limit reservoir seeding and additional resistance, in line with guideline recommendations [5, 35].

Our patient-level switch analysis is clinically instructive. Switches to INSTI-based regimens were uniformly successful and NRTI-only → PI switches also performed exceptionally well. In contrast, remaining on PI after failure had lower therapy success (73%), and NNRTI → PI achieved suppression in two-thirds. This hierarchy aligns with randomized and real-world evidence showing INSTI-anchored therapy to be the most reliable strategy after failure and supports choosing DTG- or BIC-based regimens when resistance and tolerability permit [35]. Male sex also predicted faster resuppression in our cohort (HR 1.81), whereas prior studies show inconsistent sex effects on suppression/resuppression, likely reflecting differences in adherence, pregnancy-related ART exposure, and care engagement [9, 36, 37].

Although we did not collect systematic adverse effects (AE) data, improved tolerability of modern regimens likely contributed to the era-wise decline in VF and the strong performance of INSTI-based switches. INSTIs are associated with fewer discontinuations and better tolerability than NNRTI- or PI-based anchors, in contrast to legacy regimens such as efavirenz, which frequently caused neuropsychiatric adverse events [35, 38]. The uniform success of INSTI-based switches in our cohort is consistent with this profile. While weight gain under INSTIs and injection-site reactions with LA CAB/RPV require monitoring, these rarely lead to treatment withdrawal, and patient-reported outcomes generally favor modern INSTI-based or long-acting therapies [5, 16, 18].

Given that nearly 60% of our patients reported non-adherence prior to VF, LA CAB/RPV may help selected patients once suppressed and appropriately screened. Phase 3 trials show non-inferiority to oral maintenance, while recent analyses clarify risk factors for LA failure (e.g., pre-existing RPV resistance, A6/A1 subtype, obesity). Our eligibility screen identified a subset who might benefit from this approach as part of an adherence-support package [5, 16].

Key strengths of our study include its large, multicenter design, the prospective follow-up of over two decades spanning three major treatment eras, and the availability of standardized virologic monitoring with genotypes at the time of failure. Together, these features provide a robust, real-world view of long-term ART effectiveness, durability, and resistance evolution. Limitations are those inherent to observational cohorts: residual confounding by unmeasured social determinants, reliance on self-reported or pharmacy-based adherence data, incomplete genotyping in all failures, and small patient numbers within some switch categories. These factors may have limited power to detect certain associations and prevent definitive causal inference.

In the current treatment era, VF primarily reflects adherence and healthcare access rather than regimen potency. Our findings support three priorities: (i) prevent late presentation and support people with substance use through integrated adherence services; (ii) reassess adherence and resistance promptly and switch early after confirmed VF; and (iii) favor INSTI-based salvage, or boosted PI when INSTIs are unsuitable, given their superior resuppression rates. In practice, this requires intensified outreach and education to promote earlier HIV testing, and integration of adherence and resistance checks into routine follow-up visits or structured check-up programs.

## Conclusion

In this comprehensive analysis of the RESINA cohort, VF has become rare but persists among individuals facing social, structural, and clinical barriers. Multiclass resistance highlights the need for timely genotyping and access to newer drug classes. Male sex and lower viral burden at failure were associated with faster resuppression, supporting targeted adherence support and rapid regimen optimization to sustain long-term virologic control.

## Supplementary Information

Below is the link to the electronic supplementary material.

Supplementary file1 (DOCX 44 KB)

## Data Availability

De-identified participant data are not publicly available due to privacy and institutional restrictions (GDPR). Aggregate results and the analysis code, along with a limited de-identified dataset sufficient to reproduce the main findings, are available from the corresponding author upon reasonable request.
