# Supplementary material for: HIV-1 virologic failure in the RESINA cohort: lessons from two decades of real-world data
Source: Infection. 2025 Dec 17;54(2):817–27. doi: 10.1007/s15010-025-02713-7 (PMC13021726; doi:10.1007/s15010-025-02713-7)
Supplement: Supplementary file 1 — Supplementary file1 (DOCX 44 KB) [file 15010_2025_2713_MOESM1_ESM.docx]

**Summary**

 **Supplementary Table 1a.** Multivariable logistic regression for risk factors for VF (incl. HIV-1 subtype)

 **Supplementary Table 1b.** Multivariable logistic regression for risk factors for VF (incl. region of origin)

 **Supplementary Table 1c.** Multivariable logistic regression for risk factors for VF (incl. transmitted drug resistance)

 **Supplementary Table 2.** Major transmitted and acquired resistance mutations at VF (patient-level)

## **Supplementary Table 1a.** Multivariable logistic regression of risk factors for virologic failure including HIV-1 subtype

| Variable | OR | 95% CI | p-value |
| --- | --- | --- | --- |
| Age (per 10 years) | 0.88 | 0.75 – 1.05 | 0.149 |
| Male sex (vs female/other) | 0.81 | 0.53 – 1.24 | 0.334 |
| MSM (vs heterosexual) | **0.33** | 0.20 – 0.53 | **<0.001** |
| IDU (vs heterosexual) | **1.75** | 1.01 – 3.03 | **0.046** |
| CD4 <200 cells/µl | **2.18** | 1.47 – 3.24 | **<0.001** |
| CD4 ≥200 cells/µl | 0.63 | 0.37 – 1.06 | 0.079 |
| HIV RNA <100,000 copies/ml | 0.91 | 0.54 – 1.56 | 0.740 |
| HIV RNA ≥100,000 copies/ml | 1.24 | 0.73 – 2.11 | 0.431 |
| ART start 2001–2007 | **1.44** | 0.92 – 2.27 | 0.111 |
| ART start 2008–2013 | 0.97 | 0.61 – 1.54 | 0.891 |
| Subtype: non-B (vs B) | 1.03 | 0.70 – 1.53 | 0.864 |

*_Odds ratios (ORs) with 95% confidence intervals (CIs) are shown. Reference categories were:_*

*_female/other sex, heterosexual acquisition, missing baseline CD4 or HIV RNA, and ART initiation ≥2014. N=4283, VF events=139._*

## **Supplementary Table 1b.** Multivariable logistic regression of risk factors for virologic failure including region of origin

| Variable | OR | 95% CI | p-value |
| --- | --- | --- | --- |
| Age (per 10 years) | 0.86 | 0.71 – 1.03 | 0.095 |
| Male sex (vs female/other) | 0.75 | 0.49 – 1.15 | 0.191 |
| MSM (vs heterosexual) | **0.31** | 0.19 – 0.51 | **<0.001** |
| IDU (vs heterosexual) | **1.76** | 0.99 – 3.13 | **0.052** |
| CD4 <200 cells/µl | **2.46** | 1.62 – 3.74 | **<0.001** |
| CD4 ≥200 cells/µl | 0.71 | 0.42 – 1.21 | 0.207 |
| HIV RNA <100,000 copies/ml | 0.91 | 0.52 – 1.61 | 0.749 |
| HIV RNA ≥100,000 copies/ml | 1.25 | 0.70 – 2.21 | 0.448 |
| ART start 2001–2007 | **2.17** | 1.33 – 3.55 | **0.002** |
| ART start 2008–2013 | 1.36 | 0.81 – 2.26 | 0.242 |
| Region: Western Europe (vs other) | 0.97 | 0.64 – 1.48 | 0.892 |

*_Odds ratios (ORs) with 95% confidence intervals (CIs) are shown. Reference categories were: female/other sex, heterosexual acquisition,_*

*_missing baseline CD4 or HIV RNA, and ART initiation ≥2014. N=4819, VF events=128._*

## **Supplementary Table 1c.** Multivariable logistic regression of risk factors for virologic failure including transmitted drug resistance (TDR)

| Variable | OR | 95% CI | p-value |
| --- | --- | --- | --- |
| Age (per 10 years) | 0.88 | 0.75 – 1.04 | 0.143 |
| Male sex (vs female/other) | 0.80 | 0.53 – 1.20 | 0.277 |
| MSM (vs heterosexual) | **0.32** | **0.20 – 0.50** | **<0.001** |
| IDU (vs heterosexual) | **1.74** | **1.01 – 3.01** | **0.046** |
| CD4 <200 cells/µl | **2.37** | **1.59 – 3.53** | **<0.001** |
| CD4 ≥200 cells/µl | 0.64 | 0.38 – 1.07 | 0.090 |
| HIV RNA <100,000 copies/ml | 0.99 | 0.58 – 1.69 | 0.967 |
| HIV RNA ≥100,000 copies/ml | 1.40 | 0.82 – 2.39 | 0.224 |
| ART start 2001–2007 | **1.92** | **1.22 – 3.02** | **0.005** |
| ART start 2008–2013 | 1.20 | 0.75 – 1.92 | 0.446 |
| TDR (any) | 0.71 | 0.34 – 1.49 | 0.367 |

*_Odds ratios (ORs) with 95% confidence intervals (CIs) are shown. Reference categories were: female/other sex, heterosexual acquisition,_*

*_missing baseline CD4 or HIV RNA, and ART initiation ≥2014. N=5130, VF events=139._*

**Supplementary Table 2.** Major transmitted and acquired resistance mutations in participants experiencing virologic failure

| **Pat.ID** | **Date of resistance testing** | **NRTI** | **NNRTI** | **PI** | **INI** | **Previous ART** |
| --- | --- | --- | --- | --- | --- | --- |
| **48** | 04.04.03 |  |  |  |  | NRTI: AZT, 3TC, d4T, ABC, TDF  NNRTI: EFV  PI: FPV, LPV,DRV, RTV  INSTI: RAL, DTG  CCR5-AT: MVC |
|  | 08.12.04 | M41L, M184V, T215Y |  | V82A |  |  |
|  | 19.12.05 | M41L, T215Y |  | L10V, K20R, M46I, V82A, I84V, N83D |  |  |
|  | 14.09.10 | M41L, M184V, T215Y | K101K, K103N, Y188H | L10V, K20R, M46I, V82AV, I84V, H69K |  |  |
|  | 04.10.10 | M41L, M184V, T215Y | K101K, Y188H, K103N | L10LV, K20R, L63LP, V82AV, N83D, K20R |  |  |
|  | 22.01.15 | M41LM, M184V, T215C |  |  |  |  |
|  | 26.04.18 | M41L |  |  |  |  |
| **697** | 16.06.04 |  |  |  |  | NRTI: FTC, TDF, TAF  PI: LPV  INSTII: RAL, BIC  CCR5-AT: MVC |
|  | 24.08.04 |  |  |  |  |  |
|  | 05.04.06 |  |  |  |  |  |
|  | 16.07.08 |  |  | I54V, V82A |  |  |
| **964** | 09.08.01 |  |  |  |  | NRTI: AZT, 3TC, TAF  NNRTI: EFV  PI: ATV, DRV, RTV |
|  | 23.07.08 | M184V | V90I, K101E, V106I, V179IV, G190S |  |  |  |
|  | 05.01.11 |  |  |  |  |  |
|  | 19.02.13 |  |  |  |  |  |
|  | 23.08.17 |  |  |  |  |  |
| **1022** | 26.11.04 |  |  |  |  | NRTI: 3TC, AZT, FTC, TDF  NNRTI: EFV  PI: FPV, RTV |
|  | 03.01.06 | M184V | K103N, P225H |  |  |  |
|  | 26.05.06 |  |  |  |  |  |
| **1336** | 06.02.03 |  |  |  |  | NRTI: 3TC, AZT, FTC, TDF, ABC  PI: LPV  INSTI: RAL, DTG |
|  | 15.04.16 |  |  |  | N155H |  |
| **1340** | 02.08.02 |  |  |  |  | NRTI: AZT,ABC,3TC, TAF, TDF, FTC  PI: LPV, DRV  INSTI: DTG, BIC |
|  | 31.08.11 |  |  |  |  |  |
|  | 26.05.14 | K70E | Y181C, H221Y |  |  |  |
|  | 11.03.16 | K70E | H221Y, V189I, Y181C | N88NS |  |  |
| **1362** | 03.07.03 |  |  |  |  | NRTI: AZT, 3TC, ABC, TAF  NNRTI: ETR  PI: LPV, ATV, SQV, DRV, RTV  INSTI: DTG |
|  | 19.12.06 | T215F, K219EK, K70R, L210LW, D67N, M184V | K103N |  |  |  |
|  | 30.06.20 |  |  |  |  |  |
| **1383** | 03.09.01 |  |  |  |  | NRTI: AZT, 3TC, ddI, TDF, FTC  NNRTI: NVP  PI: IDV, RTV, ATV, DRV  INISTI: RAL |
|  | 24.09.02 | M184V |  |  |  |  |
|  | 06.04.05 |  |  |  |  |  |
|  | 18.04.11 |  |  |  |  |  |
|  | 05.06.14 |  |  |  |  |  |
| **1576** | 05.02.04 |  |  |  |  | NRTI: 3TC, d4T, ABC, AZT, TDF  PI: LPV |
|  | 16.03.04 |  |  |  |  |  |
|  | 06.11.06 | M184V, T215F |  |  |  |  |
|  | 23.05.07 | M184V, T215F |  |  |  |  |
| **1595** | 23.06.05 |  |  |  |  | NRTI: 3TC, ABC, TDF, FTC  PI: FPV, RTV, DRV  INSTI: DTG |
|  | 23.01.09 |  | V179D |  |  |  |
|  | 06.11.12 |  | V179DIN |  |  |  |
|  | 30.09.13 |  | V179D, M230I |  |  |  |
|  | 25.02.14 | M184V | V179D |  |  |  |
|  | 14.02.17 |  | V179D |  |  |  |
|  | 15.05.18 |  | V179D |  |  |  |
| **1602** | 23.03.04 |  |  |  |  | NRTI: 3TC, AZT, ABC, TDF, FTC  PI: SQV, LPV, DRV, RTV  CCR5-AT: MVC |
|  | 21.12.06 | K70R | A98AG, V106IM |  |  |  |
|  | 27.06.07 |  |  |  |  |  |
| **1701** | 22.12.04  10.02.10 |  |  |  | N155H | NRTI: 3TC, d4T, ABC  PI: LPV, DRV, RTV  INSTI: RAL |
| **2175** | 23.12.03 |  |  |  |  | NRTI: AZT, 3TC  NNRTI: EFV  PI: LPV, FPV, RTV |
|  | 01.06.05 |  | K103N |  |  |  |
| **2244** | 08.10.01 |  |  |  |  | NRTI: AZT, 3TC, TDF, FTC, TAF  NNRTI: NVP  PI: DRV, RTV  INSTI: RAL, DTG |
|  | 13.03.13 | M184V | A98G, Y181C |  |  |  |
|  | 06.07.17 |  | A98G, Y181C |  | Y143CPRS |  |
| **2268** | 20.05.05 |  |  |  |  | NRTI: AZT, 3TC, ABC, TDF, FTC  NNRTI: NVP  PI: LPV |
|  | 14.02.06 | M41L, D67N, M184V, T215F | K103N |  |  |  |
|  | 21.04.09 | M41L |  | K20R, I54V, H69K, V82A |  |  |
| **2767** | 26.05.06 |  |  |  |  | NRTI: ABC, 3TC, TDF, FTC, TAF  NNRTI: RPV, NVP  PI: DRV, LPV, ATV, RTV  INSTI: EVG |
|  | 22.05.07 | L74I, Y115F, M184V | H221Y, Y181I |  |  |  |
|  | 26.02.08 | L74I, Y115F, M184V | H221Y, Y181I |  |  |  |
|  | 18.05.09 | L74IL |  |  |  |  |
|  | 03.09.12 |  |  |  |  |  |
|  | 17.01.14 |  |  |  |  |  |
|  | 25.01.17 |  | G190E |  |  |  |
|  | 26.10.17 |  |  |  |  |  |
| **4400** | 05.02.08 |  |  |  |  | NRTI: TDF, FTC  NNRTI: NVP |
|  | 04.05.15 |  |  |  |  |  |
|  | 20.01.16 | Y181C, M184I | Y181C, V179I, V189I |  |  |  |
| **10235** | 17.06.09 |  |  |  |  | NRTI: FTC, TDF  NNRTI: EFV  PI: DRV, RTV  INSTI: RAL, EVG, DTG |
|  | 19.02.11 |  |  |  |  |  |
|  | 23.05.13 |  | P236LP |  |  |  |
|  | 09.04.14 |  |  |  |  |  |
|  | 17.06.15 | M184V, K65R |  |  | E92EQ |  |
| **10418** | 01.09.09 |  |  |  |  | NRTI: TDF, FTC, 3TC, ABC  NNRTI: NVP  PI: DRV, RTV  INSTI: DTG  CCR5-AT: MVC |
|  | 17.12.09 | M184I | Y181C, M230L |  |  |  |
|  | 20.01.10 | M184I | Y181C, Y188C, M230L |  |  |  |
| **10783** | 27.11.09 |  |  |  |  | NRTI: TDF, FTC, TAF  NNRTI: EFV  PI: ATV, RTV  INI: RAL, BIC |
|  | 28.09.10 |  | K101E, E138A |  |  |  |
| **10952** | 28.01.10 |  |  |  |  | NRTI: TDF, FTC, TAF  NNRTI: EFV  INSTI: EVG |
|  | 30.10.15 |  | K103N |  |  |  |
| **11499** | 14.09.10 |  |  |  |  | NRTI: ABC, 3TC  NNRTI: NVP  PI: PV, DRV, RTV  INSTI: RAL, DTG  CCR5-AT: MVC |
|  | 10.03.11 | M184V | Y181C |  |  |  |
|  | 11.12.12 |  |  |  |  |  |
| **11744** | 17.01.11 |  |  |  |  | NRTI: ABC, 3TC  PI: ATV, DRV, RTV  INSTI: RAL, DTG |
|  | 15.09.11 |  |  |  |  |  |
|  | 30.04.13 |  |  |  |  |  |
|  | 08.03.16 | M184I |  |  |  |  |
| **11912** | 04.04.11 |  |  |  |  | NRTI: TDF, FTC, AZT, 3TC  NNRTI: NVP  PI: RTV, DRV, LPV  INSTI: DTG  CCR5-AT: MVC |
|  | 15.07.13 |  |  |  |  |  |
|  | 15.11.13 | K65R, M184V, K70K | K101K, Y181C, G190S |  |  |  |
| **12619** | 25.11.11 |  |  |  |  | NRTI: TDF, FTC, TAF  PI: DRV, RTV  INSTI: BIC |
|  | 27.01.14 | M184I |  |  |  |  |
|  | 09.10.19 |  |  |  |  |  |
|  | 06.11.19 |  |  |  |  |  |
| **12652** | 13.12.11 |  |  |  |  | NRTI: TDF, FTC  NNRTI: EFV  PI: DRV, RTV, ATV  INSTI: RAL; DTG |
|  | 27.11.12 | M41LV, M184V | K101E, V179I, G190S |  |  |  |
|  | 24.02.14 | M184V | A98AG, K101E, V179I, G190S |  |  |  |
| **13104** | 19.07.12 |  |  |  |  | NRTI: 3TC, ABC, TDF, FTC, TAF  NNRTI: NVP  PI: DRV, RTV  INSTI: DTG |
|  | 05.10.12 | L74V, Y181C, M184V | Y181C, H221Y |  |  |  |
| **13719** | 12.02.13 |  |  |  |  | NRTI: TDF, FTC, ABC, 3TC  NNRTI: ETR, EFV  PI: DRV, RTV  INSTI: RAL  CCR5-AT: MVC |
|  | 19.03.13 | A62V |  |  |  |  |
|  | 22.05.13 |  |  |  |  |  |
|  | 09.07.13 |  |  |  |  |  |
|  | 24.10.13 |  |  |  |  |  |
|  | 22.05.14 | M184I | K101E |  |  |  |
|  | 13.11.14 |  |  |  |  |  |
| **14528** |  |  | K103N |  |  | NRTI. TDF, FTC, TAF  NNRTI: EFV  PI: DRV, RTV, ATV |
| **15774** | 31.10.14 | K70R |  | V82A |  | NRTI: 3TC, AZT, TDF, TAF, FTC  NNRTI: RPV  PI: ATV, DRV, RTV  INSTI: RAL |
| **15920** | 17.04.15 | M184I | M230I |  |  | NRTI: TDF, TAF, FTC  PI: DRV, RTV |
| **17666** | 21.07.16 | K65R |  |  |  | NRTI: TAF, FTC  INSTI: DTG |
| **18613** | 16.03.17 |  | V179D |  |  | NRTI: TAF, FTC  PI: DRV, RTV  INSTI: DTG |
|  | 22.11.17 | M184V | V179D |  | R263K, G118R |  |
| **20683** | 24.09.18 |  |  | L90M |  | NRTI: TAF, FTC  INSTI: BIC |
| **22070** | 25.06.19 |  | K103HN, Y181C, G190A |  |  | NRTI: 3TC, TDF, TAF, FTC  NNRTI: DOR  INSTI: BIC |
| **22420** |  | M184V |  |  |  | NRTI: 3TC, TAF, FTC  INSTI: DTG, BIC |

*_NRTI: nucleoside analogue; NNRTI: non-nucleoside analogue; PI: protease inhibitor; INSTI: integrase inhibitor; CCR5-AT: CCR5 antagonist; ART: antiretroviral therapy; 3TC: lamivudine; AZT: zidovudine; ABC: abacavir;_*

*_d4T: stavudine; TDF: Tenofovir disoproxilfumarat; TAF: Tenofoviralafenamid; FTC: emtricitabine; NVP: nevirapine; EFV: efavirenz; RPV: Rilpivirine; DOR: doravirin; ETR: etravirine; LPV: lopinavir; ATV: atazanavir; DRV:_*

*_darunavir; SQV: saquinavir; FPV: fosamprenavir; RAL: raltegravir; EVG: elvitegravir; DTG: dolutegravir; BIC: bictegravir; MVC: maraviroc_*
